# Systematic top-down approach to clinical chemistry

**DOI:** 10.1155/S1463924693000288

**Published:** 1993

**Authors:** Harry L. Pardue, Alain Truchaud, Kyoichi Ozawa, John Place, Paul Schnipelsky

**Affiliations:** Department of Chemistry 1393 Brown Building Purdue University West Lafayette Indiana 47907-1393 USA

## Abstract

This paper introduces a systematic approach to organizing the discipline of clinical chemistry. The approach is called a top-down, systems approach because it starts at the top with the most general concepts and works down through less general concepts to the most specific details and techniques. The hypothesis is that the discipline can be organized into hierarchical levels of functional processes and operational approaches to those processes. The functional processes represent what clinical scientists do; the operatinal approaches represent how they do it. Because functional processes change little, if at all, with time, they are used to develop a stable infrastructure or framework for the discipline. That infrastructure is then used to organize and understand operational approaches that tend to change rapidly with time in response to technological advances. The paper begins with the most general functional processes and then uses selected examples of the more general functions to illustrate lower hierarchical levels or functional processes and operational approaches.


Journal of Automatic Chemistry, Vol. 15, No. 6 (November–December 1993), pp. 217–226

### Systematic top-down approach to clinical chemistry

**Harry L. Pardue*, Alain Truchaud, Kyoichi Ozawa, John Place and Paul Schnipelsky**

*Department of Chemistry, 1393 Brown Building, Purdue University, West Lafayette, Indiana 47907-1393, USA*

*This paper introduces a systematic approach to organizing the discipline of clinical chemistry. The approach is called a top-down, systems approach because it starts at the top with the most general concepts and works down through less general concepts to the most specific details and techniques. The hypothesis is that the discipline can be organized into hierarchical levels of functional processes and operational approaches to those processes. The functional processes represent what clinical scientists do; the operatinal approaches represent how they do it. Because functional processes change little, if at all, with time, they are used to develop a stable infrastructure or framework for the discipline. That infrastructure is then used to organize and understand operational approaches that tend to change rapidly with time in response to technological advances. The paper begins with the most general functional processes and then uses selected examples of the more general functions to illustrate lower hierarchical levels of functional processes and operational approaches.*

### Introduction

During this century clinical chemistry has evolved from a modest collection of routine tests to a complex and sophisticated science [1]. The needs of the medical community and the scientific and technological advances in the discipline have become increasingly diverse and complex [2]. The benefits which have resulted from this process are, of course, accompanied by some problems.

The professional roles of clinical scientists have become increasingly diverse. They include complex managerial responsibilities and maintenance of research programmes, consultation with physicians, catalysing commerical developments and evaluating new approaches to clinical instrumentation, reagent systems and procedures, as well educating clinical scientists and medical students. In addition to being responsible for laboratory services and providing consultation to medical personnel, they must oversee maintenance of good laboratory practices related to quality assurance and safety of laboratory personnel.

These changes in the professional responsibilities of clinical scientists have been accompanied by an expanding base of knowledge and technological advances that must be mastered. Advances are being made in many diverse areas, such as the chemical (immunodiagnostics and DNA probes), instrumental (microelectronics, sensors, robotics, computers), mathematical tools (chemometrics, artificial intelligence), etc. [2]. As clinical instruments have evolved from relatively simple manual devices [1] to highly-integrated, self-contained systems involving increasingly complex technologies [2], it has become increasingly difficult to identify and understand the internal functions of the systems. It has become much easier to treat these systems as black boxes, with emphasis shifted from the internal functions to external features, such as operational characteristics, throughput rates, precision, cost per test, etc.

These and other related challenges will become more severe as increasingly complex technologies are used to solve increasingly complex problems. The challenges will be compounded as such tools as robotics and artificial intelligence are used to integrate more of the analytical and diagnostic processes into self-contained systems. The best way for clinical scientists, their students, and their clients to meet these challenges is to develop and use a more systematic approach to the discipline of clinical chemistry than in the past.

The purpose of this paper is to introduce a highly structured approach to the discipline. This is called a *top-down, systematic approach* to *clinical chemistry* because topics are organized into a *systematic pattern* that begins at the *top* with the *most general concepts* and works *down* through *less general concepts* to *techniques* and *operational details* with *decreasing degrees of generality*. The heart of the approach is a general framework, or infrastructure, for the discipline that changes very little, if at all, with time. This framework is used to organize and understand those parts of the discipline, such as different technologies [2], that do change with time.

### Rationale

The primary rationale for this proposal is that the many different aspects of this or any other scientific discipline can be grouped into two general categories, namely *functional processes* and *operational approaches*. Simply stated, *functional processes* represent *what we do* and *operational approaches* represent *the ways we do it*. For example, *a separation* in a chemical determination *is a functional process*; it isolates one or more components in a sample from other components that might interfere with some other part of the process, such as the measurement step. On the other hand, *precipitation/filtration*, *liquid-liquid extraction*, *dialysis*, *liquid chromatography*, *gas chromatography*, *electrophoresis*, etc. *are all examples of operational approaches to the separation process*. In other words, a separation is a general process and the different types of separations represent the specific ways we implement this process. Other examples of functional processes include sampling, sample identification, sample processing, measurement and data-processing.

Functional processes form the general infrastructure of clinical chemistry. These processes tend to change very

* *Correspondence to Harry L. Pardue.*

© 1993 International Federation of Clinical Chemistry (IFCC).

little with time. On the other hand, operational approaches to these functions tend to be in a state of constant flux; they change with time, with technological advances and with different requirements of instrument systems, clinical procedures, clients' needs, market pressures, etc. It follows that functional processes represent a stable framework that can be used to organize and understand the more transient operational approaches and technological developments [2]. Accordingly, this paper starts with a discussion of functional processes in clinical chemistry.

### Functional processes

Functional processes can be grouped into hierarchical levels, with the highest levels being the most general and the lowest levels being the least general. Here we discuss examples from each of four hierarchical levels of functional processes in clinical chemistry.

### *Highest level*

The goal here is to identify the smallest number of functions required to encompass all the professional activities of clinical chemists. The group should not contain any redundancies, but should be sufficiently complete that any function not included can be grouped under one of the functions included in the group.

As shown in figure 1, five functions are both necessary and sufficient to satisfy these criteria. Considered in its most general and positive context, *service* is of course the primary function of the discipline, as it is with all other professions. The service function is provided through medical personnel to patients in and out of hospitals, to health maintenance programmes and in a variety of other forms. The service function is made possible by the other four functions of *research*, *development*, *education* and *management*.

It is not always possible to distinguish clearly among these functions, just as it is not always easy to distinguish clearly among different scientific disciplines. For example, in the extreme, the service function could be viewed to include other functions such as research, development and education. In other situations, it is difficult to distinguish clearly between research and development functions because these activities are frequently very closely related to one another. However, despite these areas of overlap, the meaning of each of these functions is sufficiently well understood to make them useful in a systematic organization of this or other disciplines.

### *Second level (service function)*

Each of the functions in figure 1 could be subdivided into hierarchical sublevels. However, it is beyond the scope of this paper to attempt this for all these processes. Rather, the focus is on just one, namely the service function.

Some may believe that the primary function of a clinical laboratory is to generate data for samples delivered to it. However, that is just one part of the service function in clinical chemistry. As shown in figure 2, the service function is part of a process in which the primary focus is a patient or other subject—the diagnosis and prognosis

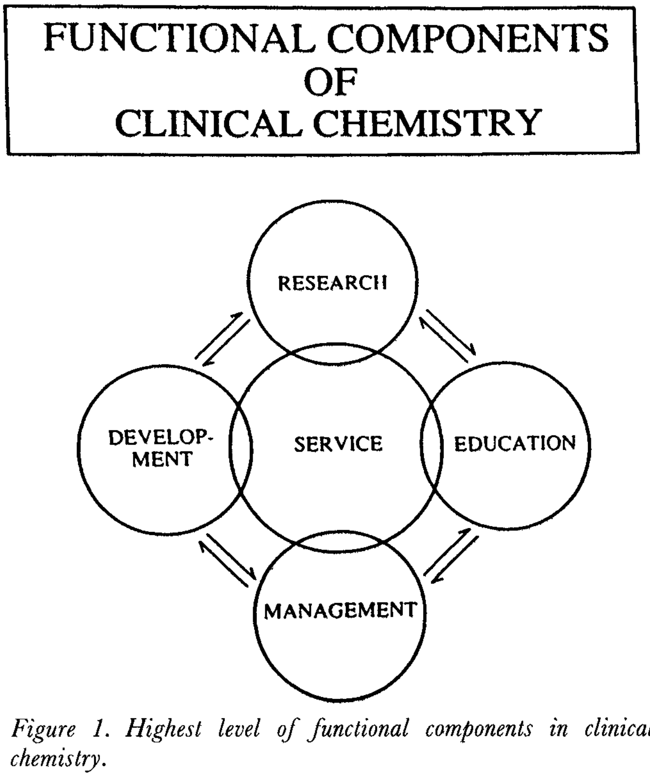

*Figure 1. Highest level of functional components in clinical chemistry.*

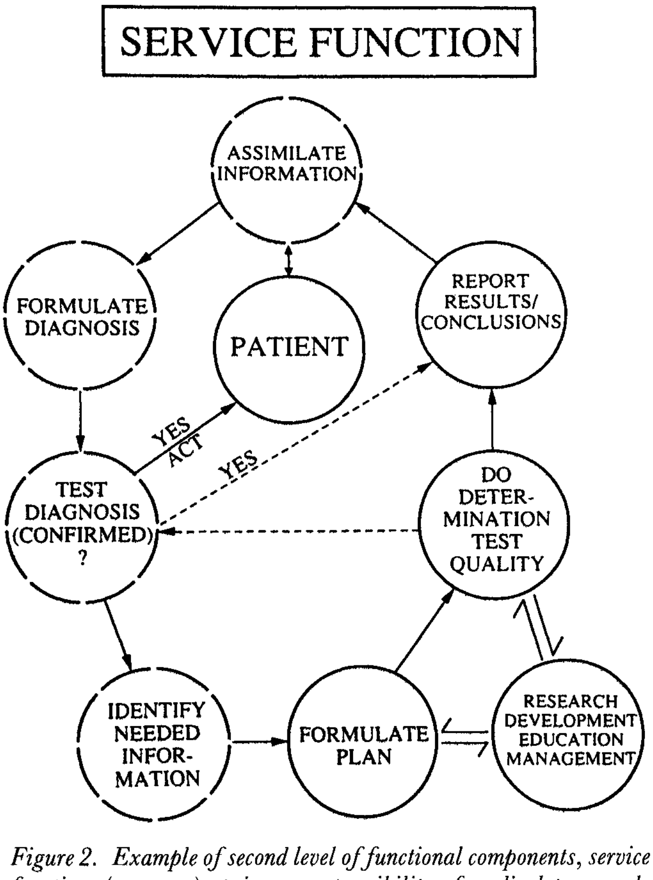

*Figure 2. Example of second level of functional components, service function.* (— — —), *primary responsibility of medical personnel;* (———), *primary responsibility of laboratory personnel.*

*of a patient's problem is a cyclic process involving interactions among medical and laboratory personnel.*

The process begins when a patient's problem is identified. The first steps in the process are to assimilate available information, formulate an initial diagnosis, test the diagnosis to determine if additional information is needed to confirm it, and, if so, to identify what additional information is needed. If chemical information is needed, then it is necessary to formulate a plan to obtain the needed information, do the necessary chemical determinations and assimilate the new information with that already available and to use it to test initial diagnoses and formulate new ones if necessary. For all but the simplest cases, this cyclic process will often be repeated several times to reach a final diagnosis and monitor the progress of a patient.

An important question is how, and by whom, the various components of the service functions in figure 2 should be handled to best serve the interests of the patient. Given the different types of expertise required to identify, determine, interpret and use laboratory results, the interests of the patient would be served best in most cases by an interactive process among medical and laboratory personnel. Although medical personnel probably should have *primary* responsibility for the functions in dashed circles in figure 2, and laboratory scientists should have *primary* responsibility for functions in solid circles, the interests of the patient will be served best if laboratory scientists (for example clinical chemists) are involved in all parts of the process for all but the simplest cases. If given sufficient information, well-qualified laboratory scientists have the background and expertise to recognize diagnostic cut-off levels or decision points, to formulate and test tentative diagnosises, and to identify additional information that will be helpful in confirming or refuting them. As indicated by the dashed arrows near the bottom of figure 2, the interests of the patient would be served best by an organization in which clinical scientists are given sufficient information and authority to test diagnoses and either report that the diagnoses are confirmed or obtain additional information if it is needed. This would significantly accelerate the diagnostic process by reducing delays between the time when results are obtained and interpreted. Artificial intelligence and expert systems could be used effectively to aid in the interpretations.

Regarding the laboratory functions themselves, for situations in which routine procedures have been developed, then the planning process may involve operational issues, such as assigning technical personnel and fitting the requested determinations into laboratory work schedules. However, for any procedure to become routine, significant amounts of advance planning must have been done. For well-established determinations, this may involve selection of appropriate procedures, reagents, instrumentation, personnel and setting up appropriate laboratory protocols. However, regardless of how established any procedure may appear, at some point in time it was not fully understood or implemented efficiently and substantial amounts of research, development and education were necessary before it could be treated as a routine procedure. Therefore, even though these features of the planning process may not be apparent in the day-to-day operation of a laboratory, their importance must not be overlooked if the newer concepts of today are to be made available as the routine procedures of tomorrow. It is ironic that the more efficient the laboratory process becomes, the less apparent the significance of the planning stage is to clients of the service.

The focus of this discussion is analytical systems, so the determination function has been chosen as the third level. The following treatment of the determination function will hopefully inspire those with interests in other parts of the service function to develop them in a systematic way.

*Third level (quantitative determination)*

The determination function in figure 3 is used here to illustrate a third level of functional processes, because this is one of the primary functions of the clinical laboratory. It is important, before discussing the functional parts of a determination, that readers note that issues such as biosafety and quality assurance must be kept in mind and acted upon throughout the process.

An important question for this paper is: what is the minimum number of non-redundant functional processes that can be used to represent all the types of operations in chemical determinations? As shown in figure 3, six different types of functional processes are necessary and sufficient to represent all the different types of processes in any type of chemical determination. These processes will not proceed in any particular order nor will all types of processes be included in all types of determinations, although most will be included in most determinations.

All determinations must begin with a *sampling* step. If a sample is physically removed from the system of interest,

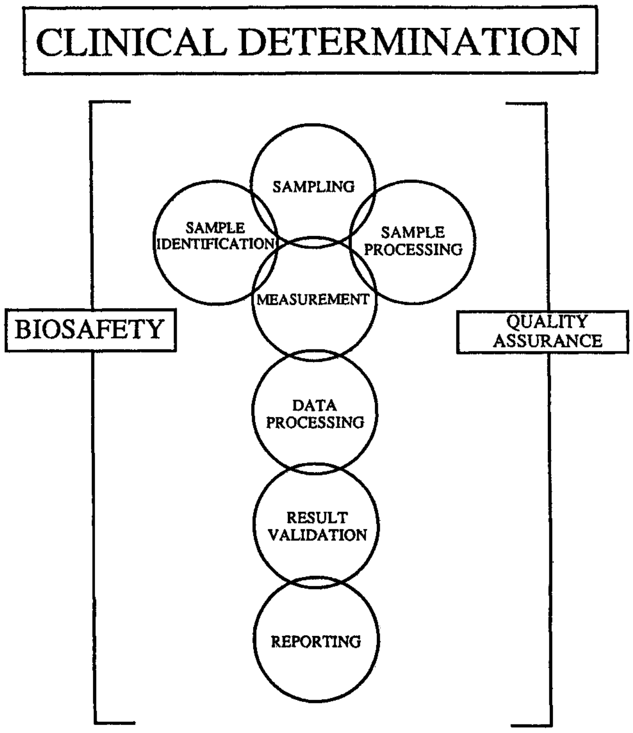

*Figure 3. Example of third level of functional processes, determination function.*

then some type of *sample identification* will be required. In most instances, some type of *sample processing* will also be needed. The sample-processing function includes such steps as addition of an anticoagulant or removal of a clot, as well as many other types of processes discussed in the next section. The function of this step is to prepare the sample for the *measurement function*, during which some *chemical or physical property* is measured. After an appropriate signal is measured, then there will usualy be some type of *data processing* required to convert measured signals to the desired information (for example analyte concentration or activity). The process is completed by a *reporting step*, in which results are conveyed in an appropriate format to the client requesting the information.

Contrary to popular usage, the *sampling* step *is a critical part of every determination.* Regardless of how carefully a sample or sample aliquot is handled after a sample is obtained, *if the sampling process is flawed the determination will be flawed.* Because it is critical that everyone associated with the sampling process understands this, this paper suggests usages which state that determinations are done on the system of interest (patients or other subjects), rather than on samples.

### *Fourth level (sample processing)*

The sample processing function in figure 3 has been selected to illustrate a fourth level of functional processes. Figure 4 illustrates five functions that can be used to describe most if not all of the different types of sample processing operations. These functions are *sample transport*, *physical changes*, *chemical changes*, *mixing* and *separation.*

If a sample is withdrawn from the system of interest (a patient or other subject), then it will be necessary to transport the sample to different locations, where the other types of operations can be done on it. These 'other types of operations' can include physical and chemical changes. *Physical changes* include changes of state and changes of physical properties, such as temperature, pressure and colour. *Chemical changes* include a wide variety of types of reactions, such as simple acid/base reactions, precipitation and coagulation reactions, enzymatic reactions, immunochemical reactions, polymerase chain reactions (PCRs) for DNA species etc. In other words, all types of chemical processes are included under the heading of chemical changes.

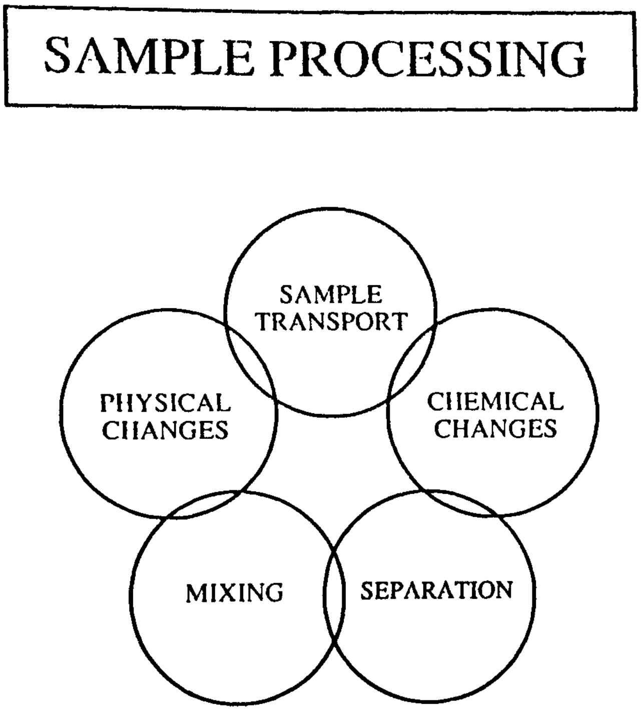

*Figure 4. Example of fourth level of functional processes, sample-processing function.*

*Mixing* is, of course, necessary to implement many different types of chemical and physical processes. *Separations* take many forms such as precipitation/filtration, dialysis, liquid-liquid extraction, many forms of chromatography, electrophoresis, capillary zone electrophoresis (CZE) and separations based on mass-to-charge ratios as in mass spectrometry etc. Again, all types of separation processes would be included under this heading.

Strictly speaking, mixing and separation are physical processes and could be included as a fifth level of functional processes. However, these processes are so prominent in so many different types of determinations that they merit inclusion at this level.

Many processes, such as precipitation, are neither strictly chemical nor strictly physical in nature. Another category, called physico-chemical changes, could be added to figure 4. However, regardless of how interrelated these processes may appear, they can all be separated into chemical processes and physical processes. Although a separate function for physico-chemical processes is not included in figure 4, it is convenient to use this term for processes that include both chemical and physical components.

Other types of functional processes in figures 1–3 could be treated in a manner similar that presented above for the service, determination and sample-processing functions. However, the goal of this paper is not a comprehensive treatment, but, rather, an illustration of the proposal for a systematic approach to clinical chemistry. Accordingly, attention is now turned to operational approaches to some of the functions described above.

### Operational approaches

Operational approaches represent the different ways functional processes are implemented. Very large textbooks would be required to describe all the operational approaches to the functions in figures 3 and 4. Accordingly, it would be hopeless to attempt a comprehensive treatment in this paper. Therefore, what is done here is to use selected examples to illustrate how a more comprehensive treatment could be organized in a systematic way.

### *Sampling function*

Figure 5 illustrates several issues that are important in the design or selection of an overall approach to the sampling function. The left-hand column in the figure

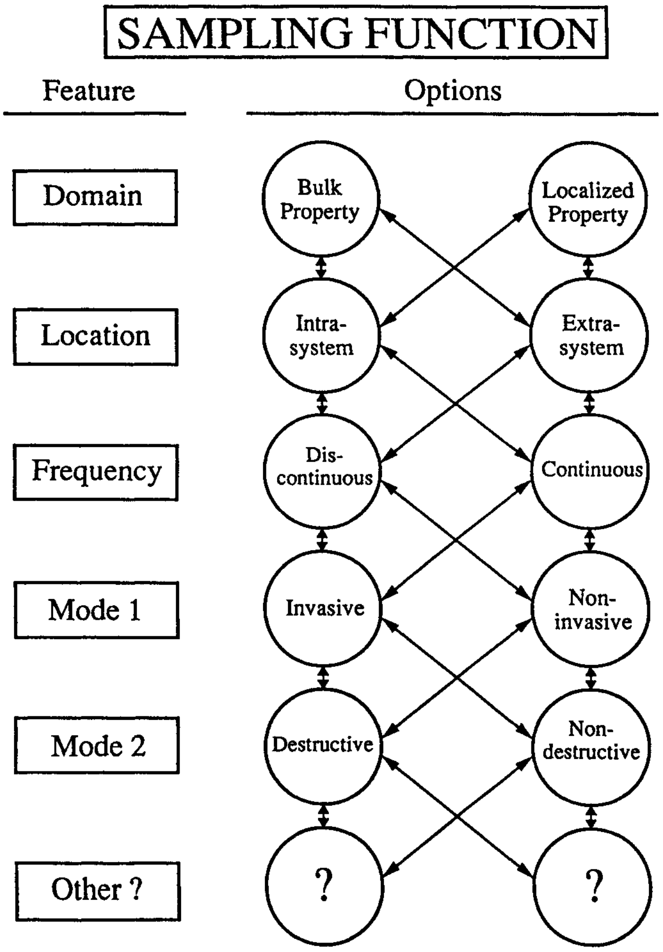

*Figure 5. Higher level operational approaches to the sampling function.*

identifies the major issues considered here. The circles on the right side of the figure identify two distinctly different subclassifiers for each of the major issues. The solid vertical and diagonal arrows identify the many different ways that these different options can be combined. Any option can be combined with any other option above or below it on either side of the figure; the only restriction is that no option can be combined with the one directly across from it.

One of the first issues that must be considered in designing a sampling procedure is what part of the system is to be sampled; this is termed the *sampling domain.* In clinical chemistry the *system of interest* is usually the human body or some part of it. However the system is defined (the body as a whole, the venous or arterial blood supply, a particular organ), there are just two choices for selecting the sampling domain. Those choices are to select the sampling domain to represent a *bulk property* of the entire system or a *localized property* of a specific part of the system. If the 'system' is defined as a particular organ, then a bulk property would be a property of the organ as a whole and a localized property would be a property of a specific part of the organ.

A second important issue is the *sampling location.* Again, there are just two choices: to process the sample inside the system (intra-system sampling); or to remove the sample from the system (extra-system sampling). In most clinical applications, samples (blood, urine, amniotic fluid) are physically removed from the patient. However, there are situations in which the sampling process takes place inside the patients. This is the case for methods such as X-ray, nuclear magnetic resonance (NMR), reflectance methods for bilirubin, microdialysis etc.; it would be the case if selective sensors were implanted in patients. In other words, the sampling process does not necessarily involve physical removal of a sample from the system of interest, although that is usually the case for most present-day methodologies.

The third important issue is the *sampling frequency*, i.e. how often samples are collected. There are two general options: to sample the system continuously (continuous sampling); or to sample the system at selected time intervals (discontinuous sampling). Usually sampling is discontinuous in chemical applications; however, with some approaches, such as implanted sensors, it could be continuous.

Other important considerations are whether the sampling process is *invasive* or *noninvasive*, and whether it is *destructive* or *non-destructive.* For lack of better terms, these options have been identified as *sampling mode 1* and *sampling mode 2.* However these issues are identified, these binary choices impose distinctly different and unique characteristics on the sampling process.

It might appear at first glance that there is redundancy between the sampling location and mode 1 options, i.e. it might appear that intra-system sampling is invasive and extra system sampling is non-invasive. However, this is not necessarily the case. For example collection of blood and urine samples are both extra-system approaches. However, although use of a syringe needle to collect a blood sample is invasive, collection of a urine sample is usually non-invasive. Analogously, intra-system sampling by implanting a sensor is invasive, whereas intra-system sampling by methods such as X-ray, NMR and reflectance measurements are not physically invasive.

Most of the sampling approaches in current use are destructive. For example, when a blood sample is withdrawn and processed in the laboratory, that is destructive. This may not be significant to an adult with a large supply of blood, but it could be very significant for an infant with a limited supply of blood.

The overall characteristics of a sampling procedure will depend upon how the different options for these and other issues are combined. As noted above, the arrows in figure 5 (and other similar figures in this section) indicate that options represented by most subclassifiers can be utilized with options represented by subclassifiers above and below them (vertical arrows) and on either side of the figures (diagonal arrows). For example, consider a path from a localized property to intra-system sampling to continuous sampling to invasive and non-destructive sampling modes. This pathway could represent the use of an implanted sensor to monitor some property (for example analyte concentration or activity) of a particular organ continuously without damaging the organ. Many other such paths could be traced to design other sampling procedures with different characteristics; the primary restriction is that no option can be used with the one directly across from it. Similar considerations apply for figures 6–8 that are discussed in the followed subsections.

*Sample-transport function*

Figure 6 illustrates several issues that should be considered in developing or selecting a *sample-transport system.* The first issue listed is implementation, namely whether the system is manual or automated. There are very few, if any, systems that are completely automated in the sense that all aspects of sample transport are automated from the point where it is withdrawn from a patient until a final result is obtained. The first parts automated were the processes after samples reached the laboratory. More recently, robots are being used to automate the transport of samples to the laboratory [3]. However, even the most recent systems still involve some manual steps to get the samples to the robots.

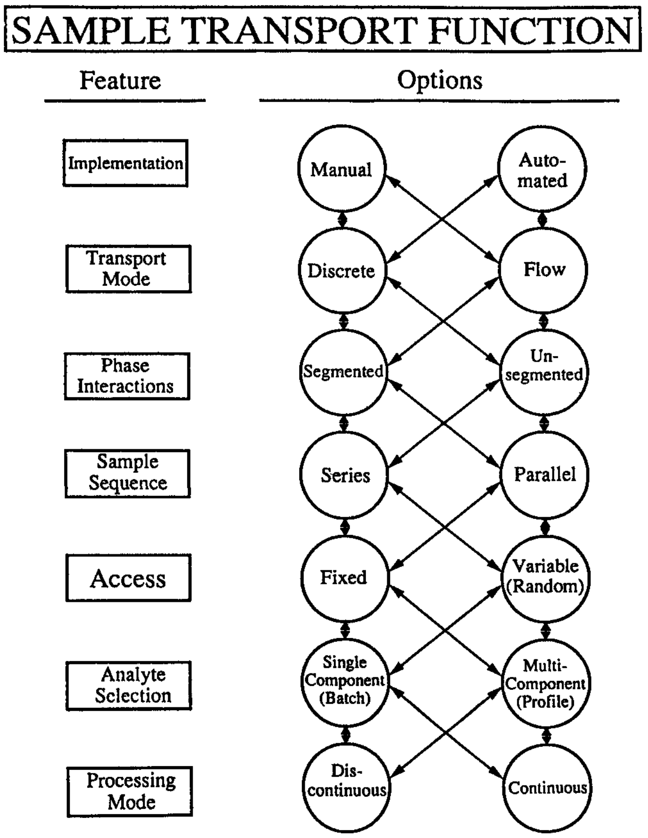

*Figure 6. Higher level operational approaches to the sample-transport function.*

The second issue involves the transport mode; the two approaches developed to date are discrete systems and flow systems. The third issue involves phase *interactions*; that is, whether samples are or are not in *segmented phases.* A primary feature of discrete-sample transport systems is that they keep samples in segmented phases, usually in individual containers; flow systems have been developed with and without segmented phases. Examples include the flow system developed by Skeggs [4] and the more recently developed approach identified as flow-injection systems [5].

*Sample sequence*, *sample access* and *analyte selection* are important issues. Samples can be *sequenced* either in *series* or in *parallel.* Most early sample-transport systems processed samples in series; more recent systems tend toward parallel processing. The question of *access* refers to whether samples are processed in a *fixed sequence*, depending on the order in which they are placed in the system, or whether the *sequence* can be *altered* with time. This latter feature is sometimes called 'random access' by analogy with computer memory. However, careful consideration of the meaning of the word 'random' (without choice or plan) shows that this term does not convey the intended meaning. 'Fixed-sequence' and 'variable-sequence' are more valid terms.

Analyte *selection* can vary from a single component (sometimes called 'batch') to multiple components (sometimes called 'profile'). Although the latter term (profile) probably is satisfactory for the intended meaning, the former (batch) conveys an entirely different concept. Specifically, one could do multicomponent determinations (profiles) simultaneously on a batch (collection) of samples.

The final issue mentioned is the processing mode, that is whether samples are moved *discontinuously* (stepwise) or *continuously* through the system. An example would be continuous-flow versus stopped-flow systems, although the concept applies equally well to discrete systems. Discontinuous operation offers more flexibility in timing sequences for different parts of the total process than does continuous operation. Discontinuous systems are advantageous for slow reactions, physical processes etc.

There probably are other issues that could be considered, but these are sufficient to illustrate the concept which is the primary goal of this paper.

In discussing the issues related to the sample-transport function this paper has not distinguished between, or focused on, features unique to any particular functional process in figure 4. The reason is that issues and choices represented in figure 6 are all relevant to all the processes in figure 4. In other words, these issues and options are just as relevant to separation methods, such as liquid-liquid extraction, chromatography and electrophoresis, as they are to the so-called 'clinical analysers' used in clinical laboratories. For example, liquid-liquid extraction is usually done in a discrete, segmented mode, while chromatography is usually done in a continuous-flow, unsegmented mode. Ion-exchange separations have been done in both discrete and flow modes, with and without segmentation. This is the beauty of the systems approach; it provides a unifying theme to many different aspects of the discripline that too often are considered as completely separate and unrelated 'techniques'.

*Measurement function*

The measurement function is considered in more detail than the other functions in order to illustrate some different levels of operational approaches. First, some of the more general issues are discussed, then some less general options are considered.

*First level*

Figure 7 shows several of the more general issues involved in the measurement function. The first issue considered is the *time* of the measurement; that is whether the measurement is a *kinetic-based* or an *equilibrium-based* method. As illustrated in figures 3 and 4, different types of processes can influence the measurement step. In fact, any of the types of processes in figures 3 and 4 can impose transient (kinetic) character on the measured signal. There is a tendency to focus more on kinetic aspects of chemical reactions than physical or physico-chemical processes. However, any or all of these processes, including the measurement system itself, can impose kinetic character on a measured signal. A measurement approach will have kinetic character if any process that influences the measured signal has not reached equilibrium when the measurement, or any part of it, is made. A measurement approach may or may not be an equilibrium-based method if all processes that influence the measurement have reached equilibrium when the measurement is made. An example of an exception would be a case in which the measured response is the time required for a process to reach equilibrium; although an equilibrium signal would be measured, the overal. procedure would represent a kinetic-based method because the result would depend on the rate at which the process approached equilibrium.

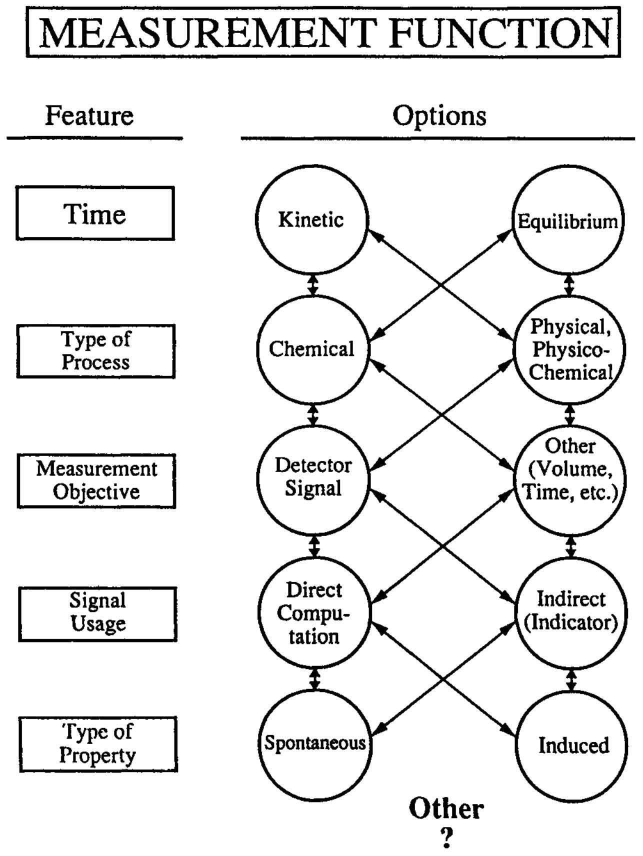

*Figure 7. Higher level operational approaches to the measurement function.*

The types of processes used can be either *chemical*, *physical* or some combination of *physico-chemical* processes.

The next issues considered are the *measurement objective* and *signal usage*, two relted concepts. Several years ago, Somogyi, realizing that the quantity measured is not always the response (signal) from a detector, introduced the concept of the 'measurement objective' [6]. The measurement objective is the quantity measured and used to compute concentration, activity or any other property of interest. In most quantitative determinations, the measurement objective is the detector signal which is used in some way to calculate concentration, activity etc. However, in some quantitative determinations such as titrations and variable-time kinetic methods [7], detector signal is used only as an indicator that some events (for example equivalence point in a titration) have occurred. Some other property, such as volume of titrant in a titration, or time interval in a variable-time kinetic method, is used to compute concentration, activity etc. The situation is reversed in qualitative determinations. In most qualitative determinations, the measurement objective is usually the position (for example wavelength or retention time) of a response rather than the amplitude of the signal. Signal amplitudes are useful, but are not the primary measurement objectives. The term measurement objective was coined as a result of Somogyi's interest [6] in what later were called variable-time kinetic methods [7]; it conveys a concept that is very useful for contemporary clinical chemistry.

The last issue included in figure 7 relates to the *type of property* measured; that is, is it *spontaneous* (for example electrochemical potential) or *induced* (fluorescence, electrolysis current etc.). This feature influences the nature of instrumentation required. Those approaches that involve spontaneous responses require only measurement circuitry; those that involve induced responses also require sources to induce the processes (for example fluorescence) that lead to measurable responses. There are, of course, other issues, such as destructive versus non-destructive approaches. However, those features included should be sufficient to illustrate the concepts at this level.

*Lower levels (kinetic methods)*

Because one of the authors has devoted a lot of effort to organizing kinetic approaches, this has been chosen to illustrate lower levels of operational approaches (figure 8).

The first issue listed is the general *shape of the response curve*. Most procedures identified as kinetic methods involve unidirectional responses (figure 9[*A*]). However, there are many kinetic (transient) responses that are not unidirectional. The most common are bidirectional (peak-shaped) responses (figure 9[*B*]) obtained from flow systems, such as both segmented and unsegmented sample processors, chromatographic systems and responses from some chemical reactions that produce unstable products [8]. All of these different responses are transient in nature and methods based on them are properly classified as kinetic methods.

The second issue involves *variable dependencies*. It is generally recognized that kinetic-based methods depend more on experimental variables than equilibrium-based methods [9]. Most methods in common use cannot compensate for these variable dependencies; these have been identified as methods *without error compensation* [10]. However, the literature contains many reports of a wide

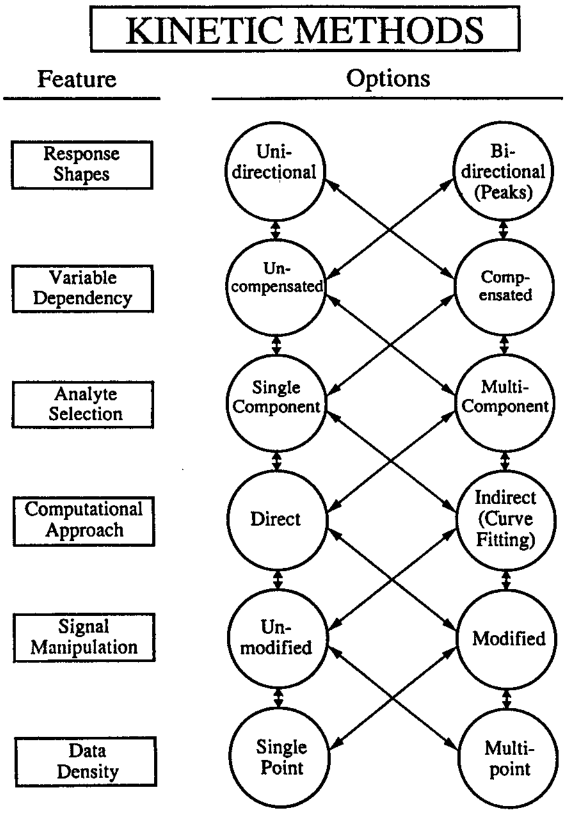

*Figure 8. Lower level operational approaches to the measurement function, kinetic methods.*

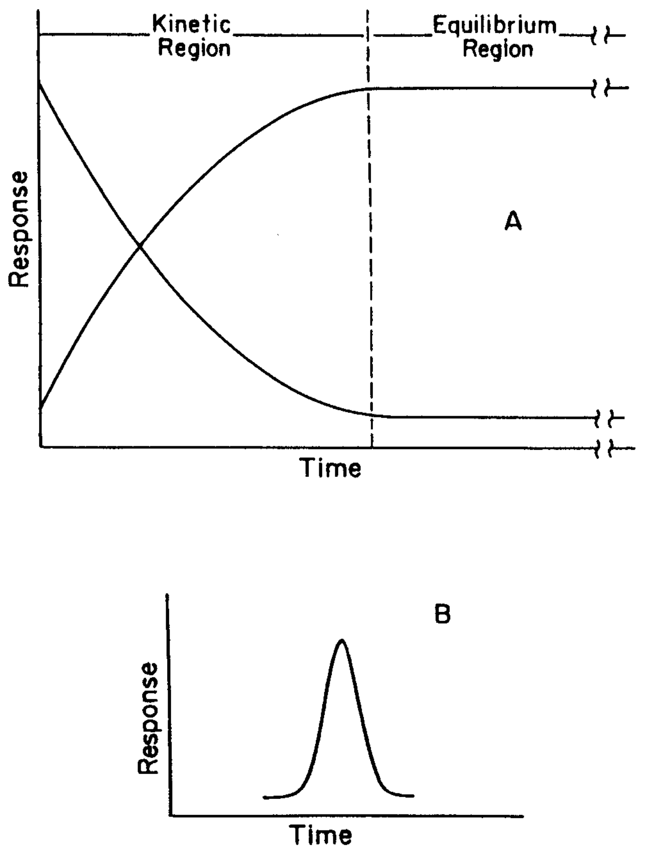

*Figure 9. Illustration of two general types of transient responses frequently found in clinical instrumentation.*

variety of methods designed specially to compensate for such dependencies [10–13]. Some of these approaches are applicable only to unidirectional signals; others are applicable to both unidirectional and bidirectional signals. This ability to compensate for changes in experimental variables, or lack thereof, is one of the most important features of kinetic methods.

The third issue, *analyte selection*, relates to whether methods are designed to quantify only one or more than one component from a single response. Although most kinetic methods are designed for single components, many different multicomponent procedures have been described in the literature [10 and 13]. Many different computational approaches have been described; most of them can be grouped according to whether concentration, activity etc., is computed directly from the measured signal, or is computed indirectly by approaches such as curve-fitting and/or extrapolation methods [10]. *Direct-computation* methods usually require detailed knowledge of kinetic behavior; *indirect curve-fitting* methods tend to be more flexible.

Response signals are manipulated in a variety of ways. One approach is to use the *signal without modification*; an example is to use signals measured at two points in time to compute the change over a fixed time period. A second approach is to *modify the signal* in some way. Examples are to compute the *rate* of change with time or to compute the *integral* of signals versus time. Options involving *unmodified signals* were most popular before the advent of readily available digital computers; options involving *modified signals* have become more popular as digital computers have become more readily available.

Data density is another issue. Characteristics of methods depend very much upon whether one, two or three or more data points are used [7].

Many other issues could be considered. However, this paper is not intended to provide a full understanding of all these aspects of kinetic methods, but, rather, to identify some of the types of issues that must be considered in lower levels of operational options. These and other aspects of kinetic methods are discussed in more detail in recent reviews [10–13] and references cited therein; in particular, these papers contain tables and related discussions in the reviews organize and extend the topics in figure 8 in much more detail.

### Discussion

It is very difficult to master a discipline as diverse and complex as clinical chemistry without some sort of systematic approach. The systematic approach has several attractive features. It differentiates clearly between functional processes that change very little with time, and operational approaches to these processes that tend to change rapidly with time. As such, it provides a stable infrastructure for the discipline that can be used in a variety of ways.

The systematic approach is versatile in the sense that it is applicable to virtually any aspect of the discipline. Topics emphasized in this paper reflect the Federation's interest in analytical systems used in the service component of the discipline. The research, development, management and education functions can also be subdivided into functional and operational components. Similarly, any one more interested in disease states than in analytical systems could organize the formulation and testing of diagnoses (see figure 2) into hierarchical levels of functional and operational components, as was done here for parts of the determination function.

The approach offers a way to dispel misconceptions that many have of the discipline of clinical chemistry. Many view the clinical laboratory solely as a place where samples go in and numerical data come out; many confuse the role of the clinical chemist with that of a technologist or technician. Many fail to understanding the need for continued research and education in the area. This is reflected in the fact that few countries have funding agencies with funds targeted specifically for research in clinical chemistry; also, very few academic institutions have educational programmes in clinical chemistry. The systematic approach proposed here can be used to demonstrate the consistencies between this and other scientific disciplines and the need for similar kinds of support.

This approach can be used, as illustrated in figure 2, to emphasize the interactive nature of the roles of medical and laboratory personnel, and to differentiate more clearly than has been done in the past between the roles of clinical scientists and laboratory technicians. It can help to formalize more active roles of clinical scientists in the diagnostic process, without undermining the roles of medical personnel. For example, referring to figure 2, the dashed line from the determination step to the testing step for a tentative diagnosis reflects the fact that most clinical scientists have the background and ability to interpret data and determine whether tentative diagnoses are valid, and whether additional data or alternative diagnoses are indicated. Clinical scientists with the freedom to exercise such judgements would be able to provide high-level information rather than just data. This could serve the interests of both medical personnel and their patients.

The infrastructure that is inherent in the approach proposed here can be useful in many ways. If one is concerned about issues such as management, quality assurance or safety, a well-conceived and organized infrastructure can help identify those areas that need attention and avoid overlooking important areas that might result from a less systematic approach. A well-organized infrastructure can help to identify the areas of the discipline in which new technologies will have their major impacts. For example, robotics will likely have its greatest impact on the sample-processing function (see figure 3). Artificial intelligence and expert systems, while having the potential to improve many aspects of instrument systems, have their most apparent impact on the diagnostic process by providing diagnotic pointers and suggesting additional information requirements and thus implementing the cyclic processes represented by figure 2.

A well-conceived infrastructure can be a tremendous aid to the education process by providing students with a well-organized structure for the learning process and by providing correlations among different topics studied. For example in the traditional approach to the study of analytical systems, analytical methods are treated more as a random collection of discrete techniques than as a well-organized pattern of interrelated concepts and approaches. This is reflected in the widespread use of terms such as calorimetric methods, spectrophotometric methods, enzymatic methods, kinetic methods, liquid chromatographic methods etc. Each of these names focuses on just one of several critical parts of every method mentioned. As illustrated in figures 3 and 4, all of these 'methods' consist of several functional processes. Simplistic names, such as liquid chromatographic methods and kinetic methods, although containing useful information, are misleading because virtually all liquid chromatographic methods involve transient responses and accordingly are kinetic-based methods. Use of these simplistic names obscures correlations and similarities among the different methods that could greatly reduce the amounts of seemingly unrelated facts that students must remember.

At a more advanced level, failure to recognize similarities such as those covered in figure 7 can represent a barrier to the extension of technical advances made in one area to other related areas. For example it has been demonstrated recently that error-compensating approaches developed for chemical kinetic processes can be adapted with similar advantages to physico-chemical processes such as electrode responses, flow systems and chromatographic signals. Rather than viewing so-called 'biosensors' [14] as some sort of isolated, unique device, the systematic approach leads one to think of these devices as conventional types of detectors with chemical reactions forced to proceed in highly localized areas near their surfaces. This realization leads one to expect that measurement/data-processing approaches used to reduce variable dependencies and extend linear ranges for analogous reactions in homogeneous solution can be used to achieve similar advantages with 'biosensors'. In short, the systematic approach can change the way one thinks about analytical systems and other parts of the clinical processes; it forces and helps one to dissect every process into its component parts and to relate those parts to their counterparts in other seemingly unrelated processes.

The *black-box syndrome* that is afflicting many in the discipline of clinical chemistry has been described. As analytical systems have become more highly integrated, the internal functions have become increasingly difficult for students and clinical scientists to understand. Virtually all of these systems are designed to implement the functions in figures 3 and 4 by using operational approaches such as those in figures 5–8. Accordingly, such a systematic approach should be useful to manufacturers who wish to describe their systems more completely, and to users of these systems who wish to understand them better. In each case, this approach provides a mechanism for a systematic way to describe and understand the different parts of complex systems.

Properly applied, the systems approach can improve communication in the area by encouraging terms which convey intended meanings. All too often, terms that do not mean what is intended are coined and popularized.

'Random access', for example, although successfully marketed, does not convey the intended meaning. Another pair of terms that does not convey the intended meaning is 'heterogeneous' and 'homogeneous' immunoassays. The correct meanings of these terms are very different than the intended meanings, namely immunoassays that do and do not involve separations. Immunoassays that involve a physical separation step are clearly heterogeneous; however, some immunoassays that do not require a separation step also have heterogeneous character. There are many such examples of poorly conceived terms that become accepted because they are never tested critically for their true meanings or consistency. Careful application of the systems approach as described herein should help those who coin terms to select terms that convey intended meanings.

Most of all, this systems approach should help to place the scientific and professional aspects of the discipline in proper perspective with its operational and technological aspects. Time, space, and other limitations have forcced this paper to focus on some very limited aspects of the total system. However, it is hoped that this effort will inspire those with greater expertise in other areas, such as the formulation and testing of diagnoses or the use of diagnostic decision points, to develop aspects of the system that were not included here or to expand and improve upon those areas discussed above.

### Acknowledgement

This study was supported in part by Grant No. GM13326-23, 24 from the National Institutes of Health.

### References

1. Somogyi, M., In Tietz, N. W. (Ed.), *Fundamentals of Clinical Chemistry* (2nd edn) (W. B. Saunders, Philadelphia, 1976).
2. Burtis, C. A. *Clinical Chemistry*, **33** (1987), 352.
3. Ozawa, K., Pardue, H. L., Schnipelsky, P., Place, J. and Trunchaud, A., *Journal of Automatic Chemistry*, **14** (1992), 9.
4. Skeggs, L. T. *Analytical Chemistry*, **38** (1966), 31A.
5. Ruzicka, J. and Hansen, E. H., *Analytical Chimica Acta*, **114** (1980), 19.
6. Somogyi, M., *Journal of Biological Chemistry*, **125** (1938), 399.
7. Pardue, H. L., *Clinical Chemistry*, **23** (1977), 2189.
8. Laios, I., Fast, D. M. and Pardue, H. L., *Analytical Chica Acta*, **180** (1986), 429.
9. Mottola, H. A. and Mark, H. B., Jr., *Analytical Chemistry*, **58** (1986), 264R.
10. Pardue, H. L., *Analytical Chimica Acta*, **216** (1989), 69.
11. Wentzel, P. D. and Crouch, S. R., *Analytical Chemistry*, **58** (1986), 2855.
12. Harris, R. C. and Hultman, E. H., *Clinical Chemistry*, **29** (1983), 2079.
13. Mottola, H. A., *Kinetic Aspects of Analytical Chemistry*, (John Wiley & Sons, New York, 1988).
14. Thompson, N. and Krull, U. U., *Analytical Chemistry*, **63** (1991), 393A.

